# Advantages of MelArray over Oncomine Focus Assay for Mutation Analysis in Melanoma

**DOI:** 10.3390/medicina62030510

**Published:** 2026-03-10

**Authors:** Andrew E. Quacoe, Sandra N. Freiberger, Mitchell P. Levesque, Reinhard Dummer, Egle Ramelyte

**Affiliations:** 1 Department of Dermatology, University Hospital Zurich and Medical Faculty, University of Zurich, 8058 Zurich, Switzerland; 2 Institute of Pathology and Molecular Pathology, University Hospital Zurich, 8091 Zurich, Switzerland

**Keywords:** skin cancer, genetic, molecular, melanoma, subtype, mutation, MelArray, Oncomine Focus Assay

## Abstract

*Background and Objectives*: Melanoma is the leading cause of skin cancer-related mortality due to its high propensity for early metastasis, although survival rates have improved with the advent of targeted and immune-based therapies. Accurate detection of targetable mutations and assessment of tumor mutational burden are essential for informed therapeutic decision-making. Mutation profiling is routinely performed using next-generation sequencing (NGS). The Oncomine Focus Assay (OFA) detects common alterations in 52 genes across various tumor entities, whereas MelArray is a melanoma-specific NGS panel covering mutations in 190 melanoma-relevant genes and providing a genome-wide copy number analysis. Moreover, tumor mutational burden is being assessed. *Materials and Methods*: In this retrospective study, we analyzed the phenotypic characteristics of 100 patients with cutaneous melanoma who underwent NGS testing using either OFA or MelArray. The aims were to compare the diagnostic yield of the two panels and to investigate potential associations between mutational profiles and clinicopathological features of melanoma. *Results*: Tumor location, ulceration, and Breslow thickness showed significant correlations with the melanoma subtypes. *BRAF* mutations were the most frequent driver alterations across all cutaneous melanoma subtypes; however, no significant correlation between specific driver mutations and phenotypic characteristics was identified. MelArray detected a notably high number of alterations in the *TERT* promoter and *CDKN2A* genes, which were not captured by OFA and are of potential therapeutic relevance. *Conclusions*: In conclusion, MelArray enables a more comprehensive molecular characterization of cutaneous melanoma compared with a small generic cancer panel and may support more personalized therapeutic decision-making.

## 1. Introduction

With approximately 2500 newly diagnosed cases annually, melanoma is the fourth most common cancer in Switzerland [1]. Although individuals of all ages may be affected, incidence peaks between 53 and 70 years of age [1,2].

Melanoma comprises several distinct subgroups, including cutaneous melanoma, acral lentiginous melanoma (ALM), uveal melanoma (UM), and mucosal melanoma [3,4,5]. Cutaneous melanoma can be further classified according to growth pattern into nodular melanoma (NM), superficial spreading melanoma (SSM), and lentigo maligna melanoma (LMM) [3,5]. ALM is considered distinct from other cutaneous melanomas due to its occurrence on glabrous skin and its characteristic mutational profile [6,7]. Additional, less common subtypes, such as desmoplastic and spitzoid melanomas, have been described and are generally classified within the cutaneous melanoma subgroup [8].

The most frequent driver mutation in cutaneous melanoma is a *BRAF* mutation, most commonly *BRAF V600E*, followed by *NRAS* mutations. Loss-of-function mutations in *NF1* represent the third most frequent genetic alteration, and melanomas lacking mutations in *BRAF*, *NRAS*, and *NF1* are commonly referred to as triple wild-type melanomas [9,10,11,12]. In contrast, ALM typically harbors fewer *BRAF* mutations and a higher prevalence of *KIT* mutations [7], while UM is characterized by alterations in *GNAQ*, *GNA11*, and *BAP1* [13,14]. Analyses of melanoma metastases have additionally revealed frequent alterations in *CDKN2A* [15,16].

At early, localized stages, melanoma can often be treated successfully with surgical excision, whereas advanced disease requires systemic therapy [8,17]. Targeted therapy is a mutation-specific treatment approach and therefore depends on the accurate identification of underlying driver mutations; established examples include *BRAF* and *MEK* inhibitors [17,18]. *TERT* promoter mutations have been associated with resistance to targeted therapies and have gained importance as prognostic biomarkers, as *TERT* inhibition has been shown to reduce tumor growth in therapy-resistant melanoma [18,19]. In recent years, immune-based therapies using checkpoint inhibitors such as anti-CTLA-4 and anti-PD-1 antibodies have become central components of melanoma treatment [20,21]. Immunotherapy may induce more durable responses than targeted therapy and has shown increased efficacy in tumors with a higher tumor mutational burden [10,21,22].

Until 2017, mutation analysis for melanoma at the University Hospital Zurich was performed using the Oncomine Focus Assay (OFA), a panel covering 52 genes, including copy number variants of 19 cancer-associated genes and 23 fusion genes [23]. Since 2018, MelArray has been implemented as a melanoma-specific next-generation sequencing panel, analyzing 190 genes frequently altered in melanoma, detecting genome-wide copy number variations, and providing additional information on HLA status and tumor mutational burden [24]. FoundationOne is another well-established, FDA-approved NGS panel, covering more than 300 genes across multiple cancer entities; however, it does not include several melanoma-specific alterations [25].

The aim of this study was to evaluate the differences between OFA and MelArray in terms of detected molecular alterations and to assess the clinical relevance of the additional information provided by a melanoma-specific sequencing panel. Furthermore, we investigated whether phenotypic characteristics of cutaneous melanoma are associated with the subtypes of cutaneous melanoma or their underlying mutations.

## 2. Materials and Methods

Patient Cohort and Data Extraction: We conducted a single-center retrospective analysis of patients with melanoma who underwent mutational analysis using either the Oncomine Focus Assay (OFA, ThermoFisher, Reinach, Switzerland) or MelArray (Zurich, Switzerland) at the University Hospital Zurich (USZ), Zurich, Switzerland. A detailed list of genes and mutations covered by both sequencing panels is provided in Table A1 in the Appendix A.

Patients were identified using KISIM, the electronic patient database of USZ, by searching for the keywords Oncomine Focus Assay, OFA, MelArray, and melanoma. This search yielded 94 patients analyzed with OFA and 292 patients analyzed with MelArray. Clinical and demographic data were extracted from electronic medical records within KISIM. Collected parameters included melanoma subtype, detected mutations, clinical stage, TNM stage according to the AJCC classification, age at diagnosis, Breslow thickness, ulceration status, tumor location, skin type, and biological sex.

Inclusion and Exclusion Criteria: Inclusion criteria were clinicopathologically confirmed cutaneous melanoma of the subtypes nodular melanoma (NM), superficial spreading melanoma (SSM), or lentigo maligna melanoma (LMM), with mutational analysis performed at USZ using OFA or MelArray between 2017 and 2022. Analyses conducted on either primary tumors or metastatic lesions were included. Exclusion criteria comprised melanoma subtypes other than NM, SSM, or LMM, melanoma of unknown primary, mutational analyses performed before 2017, or analyses conducted using panels other than OFA or MelArray.

**Figure 1:**
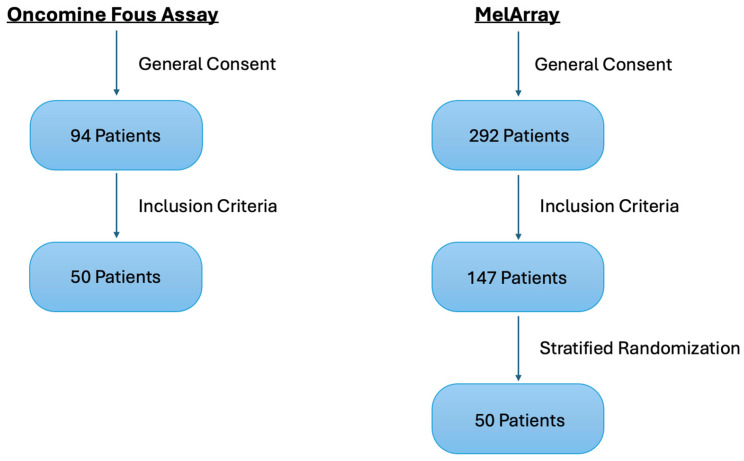
Flow diagram illustrating patient identification and inclusion in both cohorts, and stratified randomization in the MelArray group, in this retrospective single-center study.

Of the initially identified patients, 50 of 94 patients in the OFA group and 147 of 292 patients in the MelArray group met the inclusion criteria. To ensure comparability between the two cohorts, the larger MelArray group was randomized, and a stratified random selection of 50 patients was performed to match the subtype distribution of the OFA cohort. The overall study design, including patient inclusion, stratified randomization, and validation workflow, is illustrated in Figure 1.

**Figure 2:**
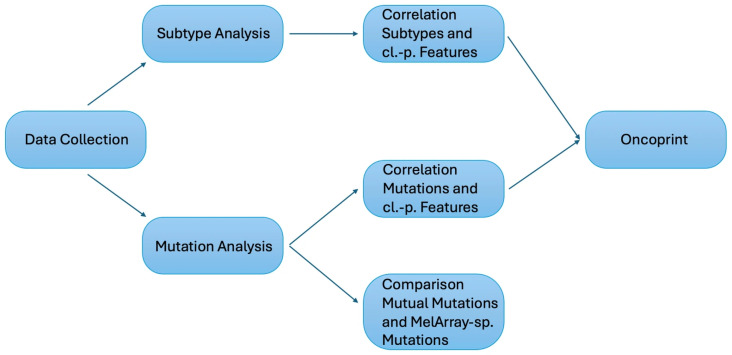
Flow diagram from the data collection process to the different analyses, statistical workflow, comparative analysis between MelArray and OFA, and generation of the Oncoprint.

The MelArray cohort would then be used separately to demonstrate the differences between mutual mutations with OFA and those solely detectable with MelArray. Subsequently, all included patients with cutaneous melanoma were combined into a single cohort (*n* = 100), irrespective of the sequencing panel used, to allow comparative analyses of the clinicopathological features and mutational patterns across panels. Only mutual mutations would be included in this step of analysis. An Oncoprint was generated using R (R Foundation for Statistical Computing, Vienna, Austria) in RStudio (Version 2026.01.1+403) with the ComplexHeatmap package to visualize mutational profiles and associated clinicopathological characteristics. Samples were hierarchically clustered based on their mutation profiles. The data collection process and analytical pipeline leading to the final dataset are shown in Figure 2.

Ethical Approval: All patients provided written informed consent. This study was approved by the Ethics Committee of Zurich (BASEC No. 2020-01344).

Statistical Analysis: Statistical analyses were performed using chi-squared tests for categorical variables, including the melanoma subtype, tumor location, and Breslow thickness categories. Adjusted standardized residuals were used for post hoc analyses where appropriate. For continuous variables such as age and Breslow thickness, Kruskal–Wallis tests were applied, followed by Dunn–Bonferroni post hoc tests. Statistical analyses were conducted using Microsoft Excel, Numiqo (DATAtab), and JASP. A two-sided *p*-value of <0.05 was considered statistically significant.

Manuscript Preparation: Artificial intelligence tools were used only to assist with grammar, scientific phrasing, and translation, and were not applied for any activities that violate ethical or legal standards.

## 3. Results

### 3.1. Patient Demographics

A total of 100 patients were included in the study cohort, comprising 50 patients analyzed using MelArray and 50 patients analyzed using the Oncomine Focus Assay (OFA). Through stratified randomization, tumor subtypes were equally distributed between the two groups, with 21 nodular melanomas, 24 superficial spreading melanomas, and five lentigo maligna melanomas in each cohort.

As shown in Table 1, 72.00% of patients were male and 28.00% were female. The median age at tumor diagnosis was 61 years, ranging from 23 to 90 years.

### 3.2. Gained Mutational Information with MelArray

Within the MelArray cohort (*n* = 50), we analyzed which of the detected mutations were shared between both sequencing panels and which were exclusively detectable by MelArray. Of the 20 distinct mutations identified in this group, 35.00% were theoretically detectable by both panels. These shared mutations included alterations in *BRAF* (70.00%) and *NRAS* (26.00%).

**Figure 3:**
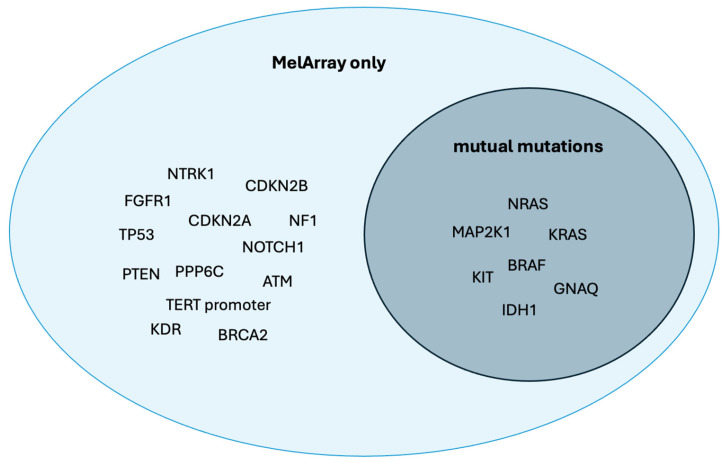
Venn diagram showing overlap of mutation coverage between only detectable with MelArray and mutual mutations in the 50 patients analyzed with MelArray. Of the 20 distinct mutations identified, 7 mutations were located within genomic regions covered by both MelArray and OFA (“mutual mutations”), whereas 13 mutations were exclusively detectable by MelArray.

Among the mutations not detectable by OFA, *TERT* promoter mutations were identified in 58.00% of patients in the MelArray cohort, while *CDKN2A* mutations were detected in 20.00% of patients. An overview of these and additional mutations identified exclusively by MelArray is provided in Figure 3.

**Figure 4:**
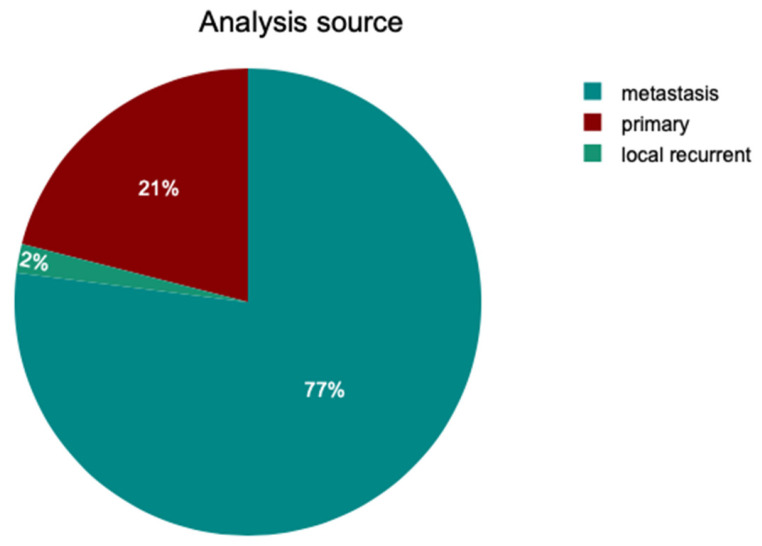
Pie chart of the source of analysis in the cohort of 100 patients (both OFA and MelArray).

In the full cohort of 100 patients, the majority of samples analyzed were obtained from metastatic sites (77.00%), while 21.00% of the samples were taken from the primary tumor. Two patients had samples collected from a local recurrence. The distribution of sample sources is illustrated in Figure 4.

Within the MelArray cohort (*n* = 50), patients were stratified according to the site of the sample collection: primary tumor (32.00%) versus metastatic site (68.00%) to evaluate the differences in detected mutations (Figure 3).

**Figure 5:**
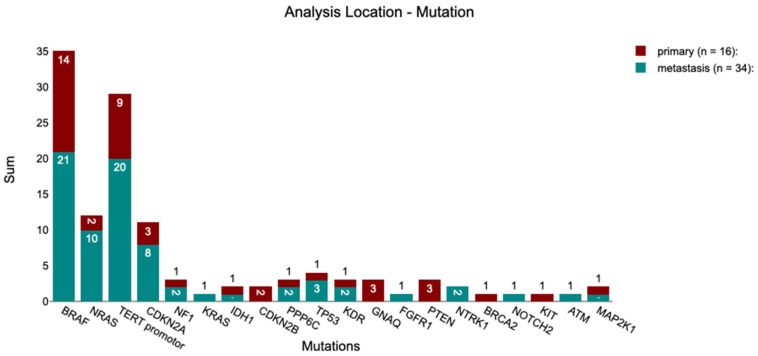
Number of tumors with the described mutations, separated in analysis made from the primary site or from a metastasis, within the group of 50 tumors analyzed with MelArray.

Among the tumors analyzed from the primary site, 87.50% of the patients harbored *BRAF* mutations, compared to 61.77% of the patients in the metastatic group. *NRAS* mutations were observed in 12.50% of the primary site samples and 29.41% of the metastatic site samples. *TERT* promoter mutations showed similar frequencies between primary (56.25%) and metastatic sites (58.82%). Additional mutations detected in each group are summarized in Figure 5.

As shown in Table 2, a total of 27 distinct mutations were identified, irrespective of whether they were detected by one or both panels. Of these 27 mutations, 51.85% were detected exclusively by MelArray, 25.93% exclusively by OFA, and 22.22% by both panels. Among the mutations detected only by MelArray, *GNAQ* would also have been detectable using OFA, while all mutations detected solely by OFA would have been detectable using MelArray (compare with Table A1 in the Appendix A). A comprehensive overview of all detected mutations is provided in Table 2.

### 3.3. Analysis of Mutual Mutations

**Figure 6:**
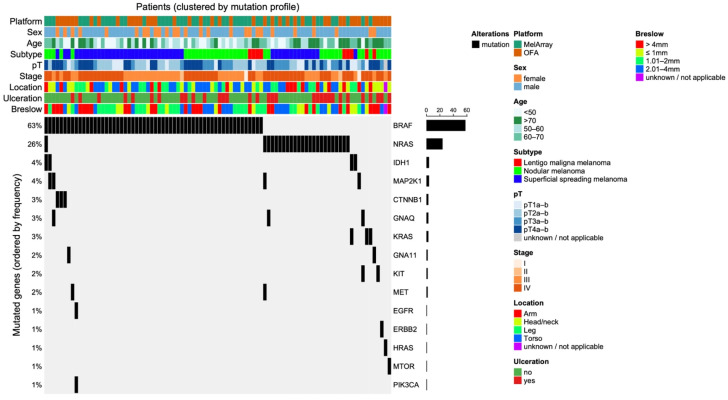
Oncoprint summarizing mutational profiles and clinicopathological characteristics (*n* = 100); only mutual mutations are included. Columns represent patients and rows represent mutated genes; black bars indicate the presence of a mutation. Genes are ordered by mutation frequency, and patients are clustered according to mutation profiles. Annotation tracks display sequencing platform, biological sex, age group, melanoma subtype, TNM classification, clinical stage, tumor location, ulceration status, and Breslow thickness category.

**Figure 7:**
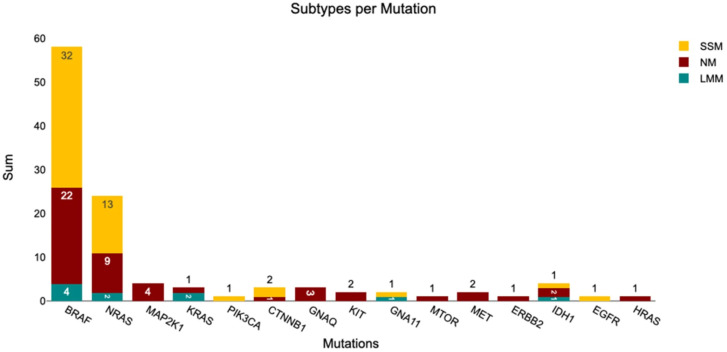
The subtypes per mutation. Only the mutual mutations of OFA and MelArray are included to enable comparability.

To enable comparability across the entire cohort (*n* = 100), we analyzed mutations detectable by both panels (mutual mutations to OFA and MelArray; see Appendix A Table A1). In total, 110 mutations were detected among the 100 tumors: *BRAF* 58.00%, *NRAS* 24.00%, *MAP2K1* 4.00%, *KRAS* 3.00%, *IDH1* 4.00%, *KIT* 2.00%, *GNAQ* 3.00%, *CTNNB1* 3.00%, *PIK3CA* 1.00%, *GNA11* 2.00%, *MTOR* 1.00%, *MET* 2.00%, *ERBB2* 1.00%, *HRAS* 1.00%, and *EGFR* (*n* = 1). Eight tumors had no detectable mutations, seven of which were analyzed with OFA. We generated an Oncoprint to create an overview of all mutual mutations in direct comparison to various clinicopathological features (Figure 6). Furthermore, mutations per subtype are summarized in Figure 7.

Among the 48 superficial spreading melanomas (SSM), 66.67% harbored *BRAF*, 27.08% *NRAS*, 4.17% *CTNNB1*, and 2.08% each of *PIK3CA, GNA11, EGFR*, and *IDH1* mutations. In the 42 nodular melanomas (NM), 52.38% had *BRAF*, 21.43% *NRAS*, 9.52% *MAP2K1*, 2.38% *KRAS*, 4.76% *KIT*, 7.14% *GNAQ*, 2.38% *CTNNB1*, 2.38% *MTOR*, 4.76% *MET*, 2.38% *ERBB2*, 2.38% *HRAS*, and 4.76% *IDH1* mutations. Of the 10 lentigo maligna melanomas (LMM), 40.00% carried *BRAF*, 20.00% *NRAS*, 20.00% *KRAS*, 10.00% *GNA11*, and 10.00% *IDH1* mutations.

#### 3.3.1. Mutations—Ulceration

Among the 35 tumors with ulceration, 45.71% harbored *BRAF* and 31.43% had *NRAS* mutations. Additional mutations in this group included *KIT* 5.71%, *GNAQ* 5.71%, and single occurrences (2.86%) of *KRAS*, *MTOR*, *MET*, and *ERBB2*. In non-ulcerated tumors of 65, 64.62% carried *BRAF* and 20.00% had *NRAS* mutations. Further alterations included *MAP2K1* 6.15%, *IDH1* 6.15%, *CTNNB1* 4.62%, *KRAS* 3.08%, and *GNA11* 3.08%, and single occurrences 1.54% of *GNAQ*, *PIK3CA*, *MET*, *HRAS*, and *EGFR*.

#### 3.3.2. Mutations—Breslow Thickness

Breslow thickness was categorized as <1 mm, 1–2 mm, 2–4 mm, and >4 mm. The distribution was 15, 33, 27, and 35 tumors in each category, respectively. *BRAF* mutations were found in 46.67%, 63.64%, 44.44%, and 51.43% tumors in these categories, respectively, while *NRAS* mutations were observed in 20.00%, 18.18%, 29.63%, and 20.00% tumors.

#### 3.3.3. Mutations—Primary Tumor Location

Primary tumor location was grouped into arm, leg, torso, and head/neck, with 14, 21, 35, and 24 tumors in each group, respectively; two tumors had an unknown primary site. Among the tumors on the arm, 50.00% had *BRAF*, 28.57% *NRAS*, and 7.14% each of *KRAS, IDH1*, and *CTNNB1* mutations. Tumors from the head/neck showed 58.33% *BRAF*, 12.50% *NRAS,* 8.33% each of *MAP2K1* and *GNA11*, and single occurrences 4.17% of *KRAS, KIT, IDH1, GNAQ, MTOR, MET*, and *ERBB2*. In the torso, 65.71% harbored *BRAF*, 22.86% *NRAS*, 5.71% each of *GNAQ* and *CTNNB1*, and single occurrences 2.86% of *MAP2K1*, *KIT*, and *MET*. Tumors from the leg showed 66.67% *BRAF*, 23.81% *NRAS*, 9.52% *IDH1*, and 4.76% each of *KRAS*, *PIK3CA*, and *EGFR*.

#### 3.3.4. Mutations—Clinical Stage

The clinical stages I, II, III, and IV were all observed in our cohort. Two tumors were classified as stage I, comprising 50.00% *BRAF* and 50.00% *MAP2K1* mutations. Stage II, likewise, included two tumors, with 50.00% harboring *BRAF* and 50.00% *NRAS* mutations. Among the 41 stage III tumors, 68.29% carried *BRAF* mutations, and 14.63% *NRAS* mutations; *MAP2K1, GNAQ, CTNNB1, PIK3CA, MET, GNA11, HRAS, EGFR*, and *IDH1* mutations were each observed once (2.44% each). Of the 55 stage IV tumors, 50.91% harbored *BRAF* mutations, 30.91% *NRAS* mutations, 5.46% *KRAS* and 5.46% *IDH1* mutations, 3.64% *MAP2K1*, *KIT*, *GNAQ*, and *CTNNB1* mutations, and *ERBB2*, *MET*, *MTOR*, and *GNA11* mutations were each observed once (1.82% each).

#### 3.3.5. Mutations—TNM Classification

Analysis of the T stage within the cohort showed that two tumors of the NM subtype were classified as Tx, as they were derived from local recurrences, and the primary tumors had already been removed prior to any analysis at the USZ. One of these two tumors harbored an *HRAS* mutation, whereas no mutation was detected in the other. Among the 14 tumors classified as T1a-b, 57.14% harbored *BRAF* mutations, 21.43% *NRAS* mutations, 14.29% *KRAS* and *IDH1* mutations each, and 7.14% *GNA11* mutations. Of the 29 tumors at stage T2a-b, 68.97% carried *BRAF* mutations, 20.69% *NRAS* mutations, 10.35% *MAP2K1* mutations, 6.90% *MET* mutations, and 3.45% *IDH1* mutations. Twenty-two tumors were classified as T3a-b, of which 50.00% harbored *BRAF* mutations, and 31.82% *NRAS* mutations; *KIT*, *GNAQ*, *CTNNB1*, *PIK3CA*, *ERBB2*, *EGFR*, and *IDH1* mutations were each observed once (4.55% each). Among the 33 tumors at stage T4a-b, 57.58% harbored *BRAF* mutations and 24.24% *NRAS* mutations; *GNAQ* and *CTNNB1* mutations accounted for 6.06% each, while *MAP2K1*, *KRAS*, *KIT*, *GNA11*, and *MTOR* mutations were each observed once (3.03% each).

#### 3.3.6. Mutations—Biological Sex

Of the 100 patients included in the study, 28.00% were female and 72.00% were male. Among the 28 female patients, 75.00% harbored a *BRAF* mutation, 14.29% a *NRAS* mutation, 7.14% a *CTNNB1* mutation, and 3.57% a *KRAS*, *GNA11*, or *MET* mutation. Among the 72 male patients, 51.39% had a *BRAF* mutation, 27.78% a *NRAS* mutation, 5.56% a *MAP2K1* mutation, and 5.56% an *IDH1* mutation. Furthermore, 4.17% had a *GNAQ* mutation, 2.78% a *KRAS* mutation, and 2.78% a *KIT* mutation, while 1.39% had a mutation in *CTNNB1*, *PIK3CA*, *GNA11*, *MTOR*, *MET*, *ERBB2*, *HRAS*, or *EGFR*. Of the 15 distinct mutual mutations detected, 40.00% were observed in female patients, whereas all (100%) were observed in male patients.

### 3.4. Correlation Phenotype and Subtype

We examined the associations between analyzed phenotypical characteristics and melanoma subtypes (nodular melanoma [NM], superficial spreading melanoma [SSM], and lentigo maligna melanoma [LMM]).

#### 3.4.1. Subtype—Breslow Thickness

**Figure 8:**
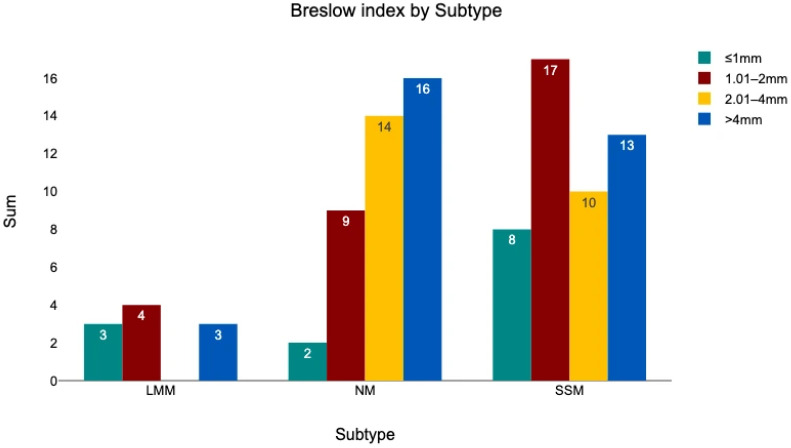
The Breslow index divided into four categories and sorted by melanoma subtype.

No significant correlation was observed between the melanoma subtype and Breslow thickness category (chi^2^ = 11.73, *p* = 0.068). A distribution of Breslow thickness across the three subtypes is shown in Figure 8. Considering exact Breslow values, the mean thickness was 4.39 mm (SD 3.87) for NM, 3.12 mm (SD 3.28) for SSM, and 2.12 mm (SD 1.87) for LMM. The Kruskal–Wallis test indicated a significant difference between subtypes based on exact Breslow values (chi^2^ = 9.29, *p* = 0.01). Post hoc Dunn–Bonferroni testing (Table 3) revealed that NM tumors had a significantly greater Breslow thickness compared with the other subtypes.

**Table 3:** Dunn–Bonferroni for the Breslow thickness between the melanoma subtypes.

Subtype Comparison of Breslow Thickness	*p*-Value *
Nodular melanoma—Superficial spreading melanoma	0.016
Nodular melanoma—Lentigo maligna melanoma	0.011
Superficial spreading melanoma—Lentigo maligna melanoma	0.268

* A *p*-value less than 0.05 was considered significant. This result indicates that NM is significantly thicker in size compared to SSM and LMM.

#### 3.4.2. Subtype—Primary Tumor Location

**Figure 9:**
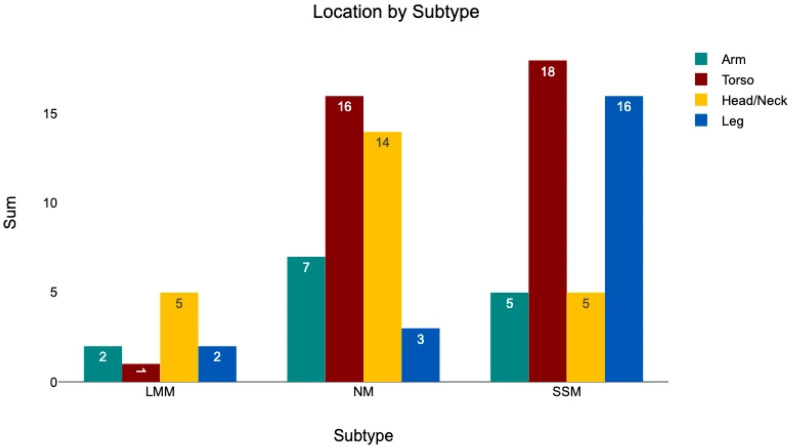
The location of the primary tumor divided into the categories arm, head/neck, leg, and torso, and sorted by melanoma subtype.

Primary tumor location was categorized as head/neck, torso, arm, or leg. The distribution of subtypes across these locations is shown in Figure 9. A significant association between melanoma subtype and primary tumor location was observed (chi^2^ = 20.18, *p* = 0.0097). Post hoc analysis using adjusted standardized residuals (z-score ≥ 1.96 indicating significant deviation from expected frequencies) demonstrated that SSM was significantly overrepresented on the legs (z = 2.91) and underrepresented in the head/neck region (z = −3.06), NM was significantly underrepresented on the legs (z = −2.90), and LMM occurred more frequently in the head/neck region (z = 2.03).

#### 3.4.3. Subtype—Ulceration

**Figure 10:**
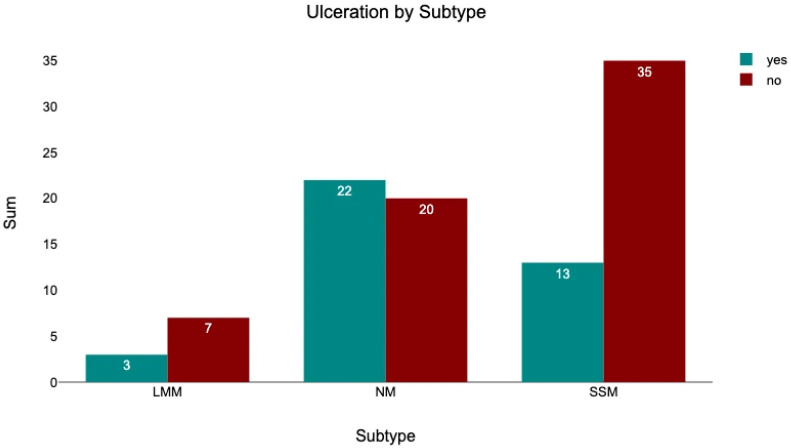
The number of patients with an ulcerated tumor, sorted by melanoma subtype.

Among the 42 NM patients, 52.38% had ulcerated tumors. In comparison, ulceration was present in 27.08% of SSM patients and 30.00% LMM patients (Figure 10). A chi^2^ test indicated a significant difference in ulceration frequency across subtypes (chi^2^ = 6.39, *p* = 0.041). Post hoc-adjusted standardized residuals revealed that NM was significantly associated with ulceration (z = 2.52), whereas SSM was associated with an absence of ulceration (z = −2.16).

#### 3.4.4. Subtype—Clinical Stage

**Figure 11:**
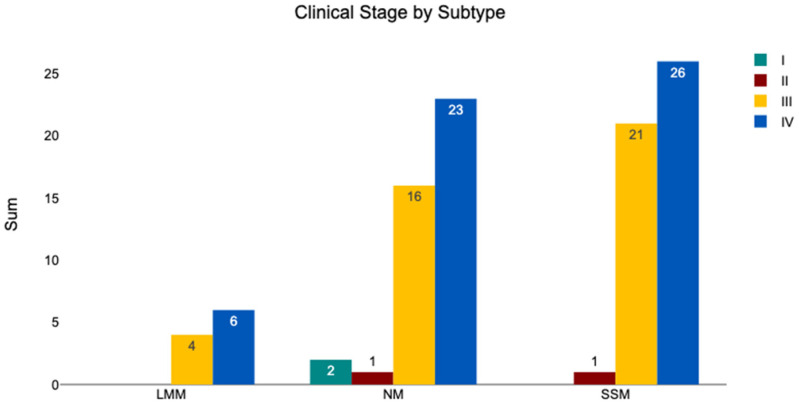
The clinical stages according to AJCC, sorted by melanoma subtype.

The clinical stage stratified by melanoma subtype is shown in Figure 11. Among the 42 NM tumors, 4.76% were classified as stage I, 2.38% as stage II, 38.10% as stage III, and 54.76% as stage IV. Of the 48 SSM tumors, none were stage I; 2.08% were stage II, 43.75% were stage III, and 54.17% were stage IV. The 10 LMM tumors comprised 40.00% stage III and 60.00% stage IV cases, with no tumors classified as stage I or II. A chi^2^ test indicated no significant difference in clinical stage across subtypes (chi^2^ = 3.22, *p* = 0.78).

#### 3.4.5. Subtype—TNM Classification

**Figure 12:**
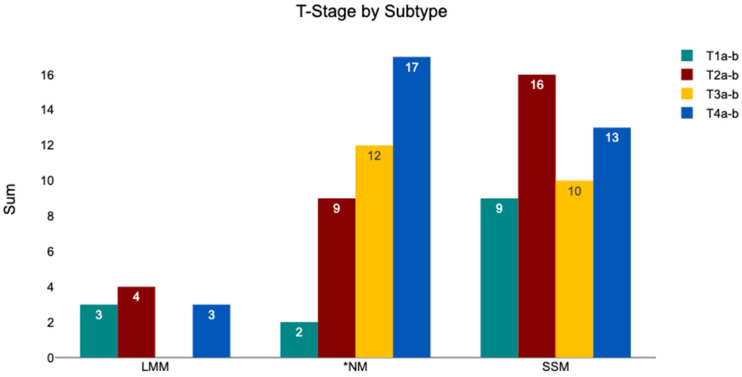
Distribution of T stages (TNM classification) stratified by melanoma subtype. * The T stage of two NM tumors was classified as Tx (not shown in this figure), with the primary tumor having been removed prior to analysis at the USZ and analysis performed from a local recurrent.

In Figure 12, the TNM stages of the tumors within the subtypes are displayed. Out of the 10 LMM patients, 30.00% were at stage T1a-b, 40.00% stage T2a-b, none at stage T3a-b, and 30.00% at stage T4a-b. Among the 42 NM tumors, 4.76% were stage T1a-b, 21.43% were stage T2a-b, 26.19% were stage T3a-b, and 42.86% were stage T4a-b. A total of 4.76% of the NM tumors were classified as Tis. Among the 48 SSM tumors, 18.75% were stage T1a-b, 33.33% were stage T2a-b, 20.83% were stage T3a-b, and 25.00% were stage T4a-b. A chi^2^ test indicated no significant difference in clinical stage across subtypes (chi^2^ = 11.47, *p* = 0.075).

#### 3.4.6. Subtype—Age at Diagnosis

The overall median age at diagnosis was 61 years (range 23–90). Stratified by subtype, the median age was 54 years for SSM, 61 years for NM, and 71 years for LMM. The Kruskal–Wallis test suggested a significant difference in age at diagnosis across subtypes (chi^2^ = 9.56, *p* = 0.008). Post hoc Dunn–Bonferroni testing (Table 4) indicated that patients with LMM were significantly older than those with NM or SSM.

#### 3.4.7. Subtype—Biological Sex

**Figure 13:**
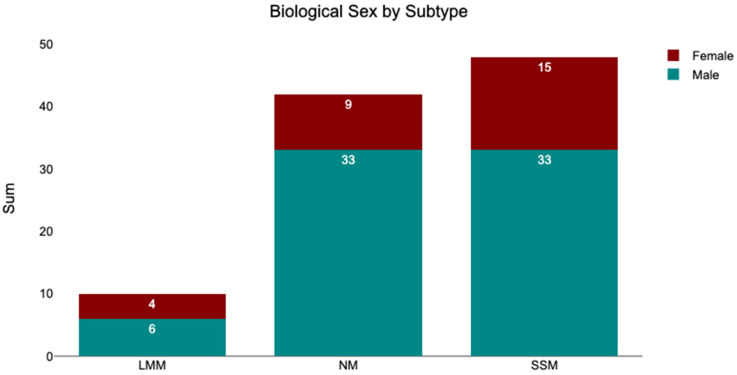
Melanoma subtypes segregated by biological sex.

As stated above, the cohort comprised 28 female and 72 male patients. Among the 10 patients with LMM tumors, 40.00% were female and 60.00% were male. Of the 42 patients with NM tumors, 21.43% were female and 78.57% were male. Among the 48 patients with SSM tumors, 31.25% were female and 68.75% were male. An overview is presented in the bar chart shown in Figure 13.

## 4. Discussion

This study demonstrates the added value of the MelArray panel in melanoma mutation analysis compared with the Oncomine Focus Assay (OFA). MelArray detects a broader spectrum of mutations, including clinically relevant alterations such as *TERT* promoter and *CDKN2A*, in addition to well-known drivers like *BRAF* and *NRAS*, which may inform personalized treatment decisions. The panel also provides tumor mutational burden (TMB), a parameter associated with responsiveness to immune therapy.

Advantages of MelArray: Only seven of the twenty mutations detected in the MelArray cohort were also identifiable by OFA, while six of those seven were indeed detected by OFA. The proportion of detected mutations that fall outside the OFA target regions highlights the diversity of mutations captured by MelArray. Moreover, all the mutations detected by OFA were also identifiable with MelArray. Because the two diagnostic methods were applied to independent patient cohorts, direct agreement statistics could not be calculated, and therefore, these results illustrate panel coverage rather than diagnostic concordance. Mutual mutations included *BRAF, NRAS, KRAS, IDH1, GNAQ, KIT*, and *MAP2K1*, which are relevant for targeted therapies [26,27,28,29,30]. *BRAF V600E* mutations can be treated with *BRAF* and *MEK* inhibitors, while *NRAS*-mutated tumors are generally managed with immune therapy, although *MEK* inhibitors may offer limited benefit. Emerging therapies, such as pan-*RAS* and *KRAS* inhibitors, as well as *IDH1* and *CDK4/6* inhibitors, show potential for tumors harboring these mutations [15,26,27,31].

Mutations detectable only by MelArray, notably *TERT* promoter and *CDKN2A*, carry important clinical implications. *TERT* promoter mutations have been associated with a worse prognosis and resistance to *BRAF* or *MEK* inhibition [18,19]. Consequently, targeting *TERT* in these resistant melanomas may potentially reduce tumor growth in *BRAF*-mutated cases that have developed resistance to *MAPK* pathway inhibition. These observations suggest that assessing *TERT* promoter status—particularly after the emergence of clinical resistance to *MAPK* inhibitors—may open additional therapeutic avenues, such as *TERT*-targeted strategies. This may represent a direct clinical advantage of MelArray over OFA.

*CDKN2A* loss-of-function mutations have been reported in approximately 40–70% of melanomas [15] and are associated with hereditary melanoma as well as a higher overall mutational burden, which may potentially enhance response to immunotherapy. *CDKN2A*-mutated melanomas may also represent a therapeutic target for *CDK4/6* inhibitors, potentially in combination with immunotherapy [15]. Notably, MelArray is capable of detecting *CDKN2A* alterations, whereas OFA is not. Given the high frequency of *CDKN2A* mutations in melanoma, this may represent a clinically relevant advantage of MelArray for personalized treatment strategies.

*NF1* loss-of-function–mutated melanomas have been associated with improved responses to PD-1/PD-L1 immune checkpoint inhibitors [32]. As *NF1* alterations are frequently observed in melanomas lacking *BRAF* and *NRAS* mutations [9], MelArray’s ability to detect these alterations may contribute to improved diagnostic and therapeutic stratification of such cases. Additionally, *NF1*-mutated melanomas may represent potential targets for *MEK* inhibition [33,34]. Given that OFA does not cover *NF1*, this may represent a further clinically relevant advantage of MelArray in the molecular characterization and personalized management of melanoma. Although this study focused on cutaneous melanoma, MelArray can detect mutations typically found in other subtypes, such as *BAP1* in uveal melanoma and *KIT* in acral lentiginous melanoma [7,14].

Furthermore, sex-stratified analyses indicated that male patients tend to have a higher number of distinct mutations than their female counterparts. Previous studies have reported a significantly higher TMB in male melanoma patients compared with female patients [35,36]. As MelArray has the capability to illustrate TMB, whereas OFA does not, these findings may further support the added value of MelArray over OFA in melanoma diagnostics and prognostics.

Mutation Patterns and Clinical Correlations: In our cohort, 58% of tumors harbored *BRAF* mutations and 24% *NRAS*, consistent with previous reports [9]. No clear associations were observed between specific mutations and phenotypical features, although *BRAF* predominance reflects its overall frequency. Comparing primary and metastatic sites, BRAF slightly decreased while *NRAS* increased in metastases, whereas *TERT* promoter and *CDKN2A* were similarly represented.

Phenotypical analysis confirmed established trends: patients with LMM were older than those with NM or SSM, and primary tumor location correlated with subtype—SSM on lower extremities, LMM on the head/neck, and NM was less frequent on legs. NM tumors were thicker and more likely to be ulcerated than SSM or LMM, consistent with previous literature [2]. These observations support the notion that phenotypical features can provide complementary information for subtype characterization, though they do not replace molecular analysis.

Clinical Implications: MelArray enables a more comprehensive molecular characterization, capturing mutations with direct therapeutic relevance as stated above, and providing TMB information to guide immune therapy. This broader coverage may enhance personalized treatment planning, particularly as targeted and immune therapies continue to evolve. While TMB was not analyzed in detail here, its availability represents a meaningful advantage, making panels that include this feature of interest for further investigation concerning melanoma analysis and treatment.

In summary, our study underscores the added diagnostic and therapeutic value of MelArray over a standard, non-melanoma-specific panel, supporting its integration into clinical practice for optimized management of cutaneous melanoma.

## 5. Conclusions

MelArray provides a broader and more melanoma-specific mutation analysis compared with the Oncomine Focus Assay, capturing clinically relevant mutations such as *TERT* promoter, NF1, and *CDKN2A* that may influence treatment decisions. The panel’s ability to report tumor mutational burden further supports personalized therapy, particularly in light of the observed sex-dependent differences in the number of distinct mutations, which may have direct implications for treatment strategies. Other phenotypical features, such as subtype, tumor thickness, ulceration, and primary location, show expected trends but cannot fully predict underlying mutations, highlighting the value of comprehensive molecular profiling. Overall, MelArray enhances the precision of melanoma characterization and may contribute to optimized, individualized patient management.

## 6. Limitations

This study has several methodological limitations. First, patients were not analyzed in parallel using both NGS panels; thus, comparisons between MelArray and the Oncomine Focus Assay (OFA) were based on theoretical detectability rather than direct head-to-head testing in the same tumor samples. Furthermore, the two panels were applied to separate patient cohorts. Allocation to OFA or MelArray was not randomized but determined by the time period of analysis, as OFA was previously the standard panel and was subsequently replaced with MelArray. This time-dependent allocation introduces potential temporal bias since evolving diagnostic strategies, staging procedures, and systemic treatment approaches over the study period may have influenced cohort characteristics.

Second, clinical data were incomplete for some patients, and information regarding subsequent systemic therapies and clinical outcomes was not included in our data collection. Therefore, we were unable to assess whether the additional molecular information provided by MelArray translated into changes in treatment decisions or improved patient outcomes.

Third, analyses were performed on both primary tumors and metastatic lesions, which may differ in their mutational profiles. This may have influenced the observed mutation frequencies.

Fourth, this study was conducted at a single center and included only cutaneous melanoma to enhance comparability between cohorts. While this approach improved internal consistency, it limits generalizability to other melanoma subtypes, such as uveal or mucosal melanoma, in which distinct mutational patterns may be present.

Finally, the overall sample size was moderate, and certain subgroups, such as lentigo maligna melanoma, were underrepresented, limiting statistical power and representativeness of subtype-specific analyses. Although MelArray enables assessment of TMB, this parameter was not systematically analyzed in the present study despite its increasing clinical relevance.

Future prospective, and ideally, multicenter studies applying both panels to the same tumor samples while incorporating comprehensive clinical outcome data, including TMB analyses, are warranted to further clarify the clinical impact of expanded mutational profiling in melanoma.

## Figures and Tables

**Table 1:** Demographics of the cohort of 100 patients.

Biological Sex *(%)*	
Male	72.00%
Female	28.00%
Age at diagnosis (median, range)	61 (23–90)
Subtypes (*n*)	
NM	42
SSM	48
LMM	10
Panel (*n*)	
MelArray	50
Oncomine Focus Assay	50

**Table 2:** Mutations * detected by either MelArray, OFA, or both panels in our cohort of 100 patients.

MelArray	OFA	Both
*NF1*	*CTNNB1*	*BRAF*
*TERT* promoter	*GNA11*	*NRAS*
*CDKN2A*	*MTOR*	*KRAS*
*TP53*	*MET*	*IDH1*
*PPP6C*	*ERBB2*	*KIT*
*CDKN2B*	*EGFR*	*MAP2K1*
*GNAQ*	*HRAS*	
*FGFR1*		
*PTEN*		
*NTRK1*		
*BRCA2*		
*NOTCH2*		
*ATM*		
*KDR*		

* All mutations are included here, and also those not mutual to both panels. This table reflects panel coverage and mutation spectrum in our cohort and does not represent a paired patient-level comparison.

**Table 4:** Dunn–Bonferroni test for age at diagnosis between the melanoma subtypes.

Subtype Comparison of Age at Diagnosis	*p*-Value *
Nodular melanoma—Superficial spreading melanoma	0.741
Nodular melanoma—Lentigo maligna melanoma	0.005
Superficial spreading melanoma—Lentigo maligna melanoma	0.002

* A *p*-value less than 0.05 was considered significant. This result indicates that patients with LMM are significantly older than patients with NM or SMM melanoma at diagnosis.

## Data Availability

The data presented in this study are available on request from the corresponding author. The data are not publicly available due to patient privacy restrictions.

## Abbreviations

The following abbreviations are used in this manuscript:

NGS	Next-Generation Sequencing
OFA	Oncomine Focus Assay
NM	Nodular Melanoma
SSM	Superficial Spreading Melanoma
LMM	Lentigo Maligna Melanoma
ALM	Acral Lentiginous Melanoma
UM	Uveal Melanoma

## Appendix A

**Table A1:** An overview of the mutations detectable by OFA, MelArray, and both panels.

Mutual Genes	AKT1, ALK, BRAF, CDK4, CTNNB1, DDR2, EGFR, ERBB2, ERBB3, FGFR2, FGFR3, GNAQ, GNA11, HRAS, IDH1, JAK1, JAK2, KIT, KRAS, MAP2K1, MAP2K2, MET, MTOR, NRAS, PDGFRA, PIK3CA, RAF1, RET, ROS1, SMO
OFA only	AR, ERBB4, ESR1, IDH2, JAK3
MelArray only	ABCB5, ACD, ACVR1C, AKAP9, AKT2, AKT3, ARID2, ARID4B, ARID5A, ASIP, ASPM, ATM, AURKA, AURKB, BAP1, BCL2, BCL2L12, BCLAF1, CCND3, CDC42, CDK6, CDKN1A, CDKN1B, CDKN2A, CDKN2C, CHD8, CXCL1, CYP1B1, CYP7B1, DCT, DCUN1D3, DDR2, DDX3X, DLG1, DNMT1, DNMT3A, DNMT3B, DPP3, DYNC1I1, E2F1, EIF1AX, EIF4A1, EP300, ETV6, EZH2, FAM58A, FANCA, FBXW7, FGFR1, FGFR4, FYN, GNA1I2, GNAS, HERC2, HLA-A, HLA-B, HLA-C, IGF2R, IQGAP1, ITGA5, JARID2, KDR, KMT2A, KMT2B, KMT2C, KMT22D, KNSTRN, MAP2K4, MAP3K1, MAP3K2, MAP3K5, MAP3K8, MAP3K9, MAPK1, MAPK3, MC1R, MITF, MLH1, MLH3, MYCN, NF1, NFKBIE, NOTCH2, NTRK1, OCA2, PARP1, PCDHGA1, PIK3C2A, PIK3C3, PIK3CB, PIK3R1, PIK3R4, PIKFYVE, PKD2, PLA2G6, PLCB1, PLCE1, PLEKHGA, PPP2R2B, PPP2R5C, PPP3CA, PPP6C, PRAME, PRKAR1A, PRKCD, PROS1, PTCH1, PTEN, PTPN11, PTPRF, PTPRJ, PTPRK, RAC1, RAD51B, RASA1, RASA2, RASA3, RB1, RICTOR, RQCD1, SETD2, SF3B1, SHOC2, SLC1A4, SLC45A2, SMARCA4, SOS1, SOS22, SPRED1, SPRED2, SPRY4, SRC, STK11, TAOK1, TAOK2, TERF2, TERF2IP, TERT, TNRC6B, TP53, TRRAP, TSC1, TSC2, TUSC3, TYR, TYRP1
